# An In Vitro Comparison of the Influence of Air Abrasion, G-Multiprimer, and Salivary Contamination on the Shear Bond Strength and Surface Characteristics of Opaque Zirconia Ceramics

**DOI:** 10.1155/ijod/3644666

**Published:** 2025-06-16

**Authors:** Alisha Ono Idris, Shobha J. Rodrigues, Puneeth Hegde, Thilak Shetty, Umesh Y. Pai, Sharon Saldanha, Mahesh M., Sandipan Mukherjee, Ann Sales, Vignesh Kamath, Prashant Bajantri

**Affiliations:** Department of Prosthodontics, Manipal College of Dental Sciences Mangalore, Manipal Academy of Higher Education, Manipal 576104, Karnataka, India

**Keywords:** air abrasion, G-multiprimer, resin cement, saliva contamination, zirconia

## Abstract

**Aim:** To evaluate the effect of various surface treatments and saliva contamination on the shear bond strength (SBS) and surface characteristics of yttria-stabilized tetragonal zirconia ceramics (YTZP) bonded with self-adhesive resin cement (SAC).

**Materials and Methods:** Opaque Y-ZTP ceramics were divided into four groups based on specific surface treatment: control (C), air abrasion (AA), G-multiprimer (G), and AA + G (AG). Surface characteristics of the treated specimens, roughness and, elemental analysis were done using scanning electron microscopy (SEM), atomic force microscopy (AFM), and energy dispersive analysis of X-rays (EDAX), respectively. These specimens were further divided into saliva (S) and non-S (NS) contamination groups. Following the surface treatment, 10 resin cement cylinders were affixed onto the zirconia discs within every group. Micro-SBS (µSBS) was calculated, and fracture surfaces were assessed. Statistical analysis was done using one-way analysis of variance (ANOVA) and the post hoc Tukey test.

**Results:** The µSBS values for the groups were as follows: CS (0.86 ± 0.7), CNS (3.13 ± 2.7), AS (3.14 ± 2.6), ANS (2.96 ± 1.2), GS (5.92 ± 2.2), GNS (5.94 ± 2.2), AGS (4.97 ± 0.9), and AGNS (8.06 ± 1.4). A one-way ANOVA showed that all the groups had a statistically significant difference in the SBS except the AS, AGS, CNS, ANS, as well as the GNS and AGNS. AFM results revealed the mean roughness value of AA is highest, followed by G-multiprimer, AG, and least in control. This difference is statistically significant with *p*-value of <0.001. This also correlated with the SEM images.

**Conclusion:** Airborne particle abrasion and the application of G-multiprimer provide the best results in a moisture-free environment.

## 1. Introduction

The growing demand for restorative materials that combine superior esthetics, biocompatibility, and enhanced mechanical performance has driven the development of advanced ceramics, such as densely sintered aluminum oxide and zirconium oxide ceramics. These robust ceramics are widely utilized in clinical applications, including posts, fixed dental prostheses, implant abutments, and resin-bonded fixed dental prostheses [1]. Furthermore, the advent of computer-aided design and computer-aided manufacturing (CAD/CAM) technologies has significantly expanded the use of zirconia in dentistry. While CAD/CAM systems enhance precision by overcoming limitations inherent in traditional lost wax techniques, factors such as the sintering process, scanning accuracy, processing of geometric data, calculation of milling parameters, and milling itself influence the fitting accuracy of zirconia restorations [2, 3]. This often results in a larger internal gap in CAD/CAM ceramic restorations compared to conventional PFM crowns [4–9], although conflicting results are presented in the literature [10–13].

An increased cement thickness due to a larger internal gap can lead to water absorption, hydrolytic degradation of resin cements, reduced mechanical properties, residual tensile surface stresses, damage to veneering porcelain, and poor adhesive bond strength [6, 14–17]. Establishing a reliable adhesive bond between zirconia and resin cement necessitates meticulous technique and material selection [18, 19]. Consequently, pretreatment is conducted to enhance the bonding strength of the resin cement to both the restoration surface and dental tissues [20–22].

Numerous methods, including air-abrasion with Al_2_O_3_ particles, tribochemical silica coating, Er:YAG laser application, primers, combinations of surface treatments, selective infiltration techniques, plasma spraying, and silica nanofilm deposition, have been explored to improve and stabilize the bond to resin cement [21–25]. However, airborne particle abrasion has specific drawbacks, such as sharp scratches, cracks, grain pullout, material loss, and t–m phase transformation (tetragonal to monoclinic), which reduce mechanical properties [26–28].

Studies demonstrate that appropriate combinations of surface treatments, ceramic primers, and bonding agents can modify the surface characteristics of zirconia and increase bonding values [29, 30].

The introduction of 10-MDP and acid-functionalized self-adhesive resin cements (SACs) has notably improved bonding strength while reducing chairside time. SAC also exhibits superior mechanical properties compared to traditional luting agents. Its lower pH (2–3) facilitates etching and adhesion to tooth substrates, forming a stable interface between the methacrylate network and tooth tissues [31–33].

Additionally, literature suggests that incorporating γ-methacryloxypropyltrimethoxysilane (γ-MPTS) into universal adhesives can enhance the bond strength with ceramic materials. While some researchers argue that γ-MPTS exhibits limited bonding efficacy with glass ceramics due to factors, such as moisture, low pH, and early hydrolysis, other studies show that its combination with 10-MDP significantly improves bonding performance. Certain findings also highlight that residual solvents in adhesive layers and water-induced degradation of 10-MDP after thermal cycling may compromise long-term bond strength [34, 35].

Salivary contamination during restoration placement presents challenges, as it may reduce bond strength with SAC [36, 37].

To address this, novel primers have garnered attention for improving resin cement adhesion [38].

However, limited research exists regarding the impact of these primers on the long-term bonding strength of zirconia surfaces when paired with SAC.

Consequently, this study aims to evaluate the effects of various surface treatment techniques on the shear bond strength (SBS) and surface characteristics of zirconia dental ceramics bonded with SAC. While air abrasion (AA) followed by primer application remains a widely accepted method for enhancing bond strength, this investigation uniquely assesses salivary contamination's impact on opaque 3Y-TZP zirconia. Despite newer zirconia generations, opaque zirconia remains clinically relevant due to its distinctive optical properties and historical applications. The null hypothesis states that surface treatment strategies and salivary contamination will have no significant influence on zirconia's surface characteristics or µSBS to SAC.

This in vitro study was conducted in collaboration with the Department of Dental Materials and the Central Instrumentation Facility after obtaining clearance from the Institutional Ethics Committee (IEC Protocol Reference Number: 19103).

### 1.1. Sample Size Determination

At the time this study began in 2020, no prior research had explored the application of G-multiprimer and its effects on the SBS and surface characteristics between zirconia dental ceramics and SAC. To address this gap, a pilot study was undertaken to calculate the required sample size. Using a 99% confidence interval, an anticipated standard deviation of 1.208 units from pilot data, and an acceptable margin of error of 1 unit for the true population mean, the sample size calculation indicated the need for 9.71 specimens. Based on similar studies and the pilot data, the sample size was rounded to approximately 10 specimens per group [28].

## 2. Materials and Methods

This section is organized under the following headings:

  Preparation of zirconia specimens and surface treatment
  Priming agent and application
  Preparation of bonding specimens
  Thermocycling protocol
  µSBS test
  Mode of failure analysis
  Scanning electron microscopy (SEM) and energy dispersive X-ray analysis (EDAX)
  Atomic force microscopy (AFM) assessment

### 2.1. Preparation of Zirconia Specimens and Surface Treatment [36]

Zirconia discs were fabricated according to ISO 6872:2015 standards for dental ceramic materials. Twenty-eight opaque 3Y-TZP zirconia discs (18 mm in diameter and 4 mm in thickness) were milled from fully sintered, isostatically pressed blanks (Jyoti Ceramic Industries Pvt. Ltd., Maharashtra, India). Twenty-four discs were designated for SBS analysis, with each disc providing four samples that were randomly distributed into four groups based on their surface treatment. An additional four discs were reserved for SEM analysis of the surface modifications. Each major group was further divided into two subgroups based on the presence or absence of salivary contamination, resulting in a total of eight groups with 10 samples in each (*n* = 10).

The groups were defined as follows (*n* = 10):

  Group 1 (C–S): Control with saliva
  Group 2 (C–NS): Control, no saliva
  Group 3 (AA–S): Air abrasion with saliva
  Group 4 (AA–NS): Air abrasion, no saliva
  Group 5 (G–S): G-multiprimer with saliva
  Group 6 (G–NS): G-multiprimer, no saliva
  Group 7 (AG–S): Air abrasion + G-multiprimer with saliva
  Group 8 (AG–NS): Air abrasion + G-multiprimer, no saliva

C–S and C–nonsaliva (NS) did not undergo any further surface modification treatment. For AA–S and AA–NS, each disc was subjected to AA performed using 110-µm aluminum oxide particles at 4 bars for 15 s, from a 10-mm distance and a 45° angle [28]. Although some literature advocates for lower pressures (2.5 bars) and smaller particle sizes (50 µm) to reduce surface damage, our pilot tests indicated that these parameters optimized surface roughness specifically for opaque 3YTZP without compromising its mechanical integrity. Following the sandblasting process, the discs were cleaned using an air syringe.

For the primer-only groups G–S and G–NS, zirconia specimens were first cleaned in an ultrasonic bath with isopropanol, rinsed, and air-dried. A thin layer of G-multiprimer was then applied using a microbrush. In the combined treatment groups (AA + G [AG]–S and AG–NS), specimens underwent the same AA process followed by cleaning and primer application.

Fresh saliva collected from the healthy, nonalcoholic, and nonsmoking primary author after a 90-min fasting period, following established protocols [36]. Saliva was applied onto the zirconia discs using a microbrush for 10 s [36]. The saliva was then allowed to remain on the surface for exactly 60 s to ensure uniform film formation, after which resin cement was applied immediately.

### 2.2. Priming Agent and Application

G-multiprimer (GC Corporation, Tokyo, Japan) is a primer formulated with γMPTS, 10MDP, MDTP (methacryloyloxydecyl dihydrogen thiophosphate), BisGMA (bisphenol-A glycidyl methacrylate), TEGDMA (triethylene glycol dimethacrylate), and ethanol. Following the cleaning of the zirconia surfaces, the primer was applied using a microbrush in strict adherence to the manufacturer's protocol to optimize bonding.

### 2.3. Preparation of the Bonding Specimens [28]

Eighty samples of Rely-X cement pillars (Rely X U 200, 3M Company, St. Paul, MN, USA) were fabricated on the pretreated opaque zirconia discs. Ten Tygon tubes (each with an internal diameter of 0.8 mm and a height of 5 mm) were prepared and allocated among three discs per group. Four tubes were applied to each disc, with two additional tubes planned to account for potential fractures during processing. Resin cement was dispensed, mixed, and loaded into each Tygon tube before being positioned on the disc surface at 5 mm intervals. The samples were then light-cured for 30 s (following the manufacturer's instructions) and allowed to incubate for 24 h at room temperature to ensure complete polymerization. Finally, the Tygon tubes were carefully removed using a sharp blade.

### 2.4. Thermocycling Protocol

Each specimen underwent thermocycling between water baths maintained at 5 and 55°C with a dwell time of 15 s per cycle, for a total of 5000 cycles. This protocol approximates 6 months of intraoral thermal stress as supported by current literature [39].

### 2.5. µSBS Test

The µSBS test was conducted using a universal testing machine (Zwick/Roell Z020, Ulm, Germany). A blade applied a loading force precisely at the junction where the resin cement pillar met the zirconia disc. The disc was positioned perpendicularly to the blade using a heat-cured polymethylmethacrylate block (dimensions: 40 × 20 × 25 mm). A shear load was applied at a crosshead speed of 0.5 mm/min until failure occurred. The peak load at failure (measured in Newtons, N) was divided by the cement's surface area (in mm^2^) to calculate the SBS expressed in megapascals (MPa).

### 2.6. Mode of Failure Analysis

Immediately following the SBS test, the debonded zirconia surfaces were examined using a compound zoom microscope (Olympus, Olympus Scientific Solutions America Corp, PA, USA) at 40× magnification. Failure modes were classified as follows: adhesive (at the resin cement/zirconia interface), cohesive (within the resin cement or zirconia), or mixed (a combination of adhesive and cohesive failures). Three independent observers evaluated all samples to minimize observer bias.

### 2.7. SEM, Surface Elemental Analysis

Representative discs from each main group were randomly selected for surface micromorphology evaluation using SEM (Zeiss EVO MA 18, Carl Zeiss, Jena, Germany). Prior to analysis, each sample was sputter-coated with a 10 µm-thick gold layer. Following sputter coating, SEM was employed to analyze the pretreated surfaces across all groups. Complementary EDAX (Oxford EDS [X-act], Abingdon, United Kingdom) was used to determine the elemental composition of the differently treated specimens.

### 2.8. AFM

To further assess the surface roughness and topographical characteristics of ceramics, representative discs (previously analyzed via SEM) were subjected to AFM (Innova SPM AFM, Bruker, MA, USA). A noncontact method was employed using an AFM cantilever equipped with magneto-resistive sensors. Three random locations were scanned on each disc over an area of 50 × 50 µm. The arithmetic average roughness (Ra), root mean square roughness (Rq), and maximum peak-to-valley roughness (Rmax) were recorded in nanometers.

### 2.9. Statistical Analysis

Data were analyzed using SPSS Statistics software (IBM, version 20, Chicago, USA), with results achieving statistical significance at *p*  < 0.05. An independent *t*-test was employed to compare each technique between the saliva-contaminated and NS groups. For the µSBS results, one-way analysis of variance (ANOVA) was performed, followed by the post hoc Tukey HSD test to assess pairwise differences. Additionally, a chi-square test was conducted to evaluate differences in the mode of failure among all groups.

## 3. Results

**Figure 1:**
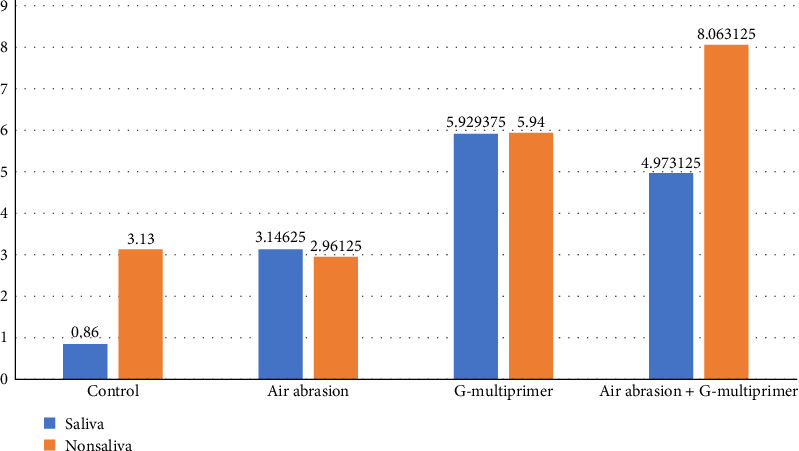
Comparison of saliva and nonsaliva groups in each of the techniques using independent *t*-test.

**Figure 2:**
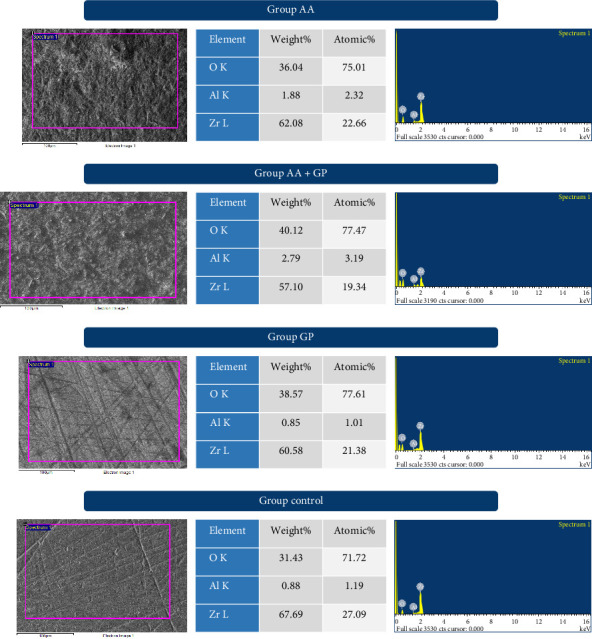
SEM and EDAX images of air abrasion group (AA), air abrasion + G-multiprimer group (AG), G-multiprimer group (G), and control (D).

**Figure 3:**
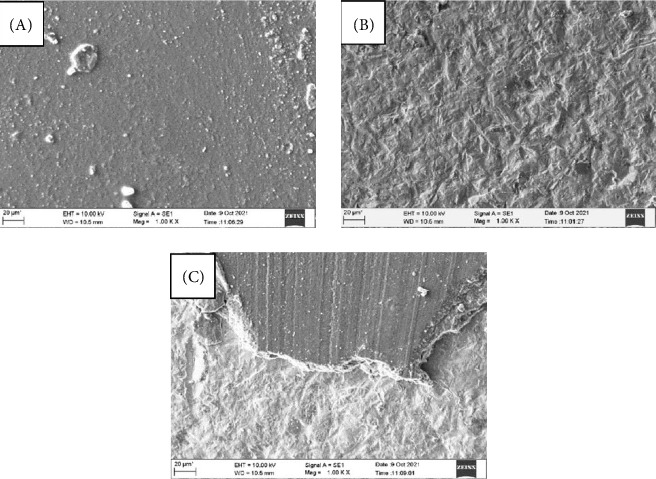
SEM images reveal the mode of failures: (A) adhesive failure, (B) cohesive failure, and (C) mixed failures.

**Figure 4:**
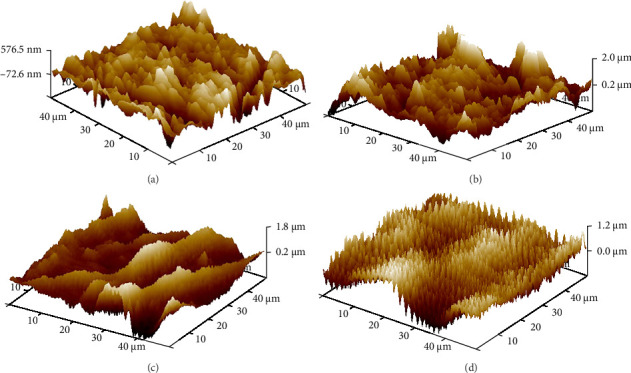
AFM 3D Images of images of control group (A), air abrasion group (B), G-multiprimer group (C), and air abrasion + G-multiprimer group (D).

Results are presented in Tables 1–3 and Figures 1–4.

### 3.1. SBS of the SAC

According to the results, surface treatment of zirconia significantly improved the SBS compared to the control (*p* < 0.05). Comparison of the SBS amongst C–S and C–NS shows that SBS is higher in NS group with a *t*-value of −2.536 and is statistically significant (*p*=0.029). Comparison of SBS amongst saliva-contaminated groups using a way ANOVA test shows that the mean value of G–S (5.92 MPa) is highest followed by AG–S (4.97 MPa), AA–S (3.14 MPa) least in C–S (0.86 MPa). This difference is statistically significant with a test value of 14.761 and *p*-value of <0.001. Comparison of SBS amongst NS contaminated groups using one-way ANOVA test shows that the mean value of AG–NS (8.06 MPa) is highest, followed by G–NS (5.94 MPa), C–NS (3.13 MPa) least in AA–NS (2.96 MPa). This difference is statistically significant, which showed the test value of 19.306 and *p*-value of <0.001. Post hoc Tukey test shows that the difference between control and AA is not statistically significantwith a mean difference of 0.16875 and *p*-value of 0.998. Comparison of the SBS between the two combinations shows that SBS is higher in AG–NS with a *t*-value of −5.447 and is statistically significant with a *p*-value of <0.001 (Figure 1).

### 3.2. Micromorphology Analysis (Figures 2 and 3)

SEM images, captured at 200× and 1000× magnifications, revealed distinct surface features across the groups. In the AA group, the impact of alumina particles produced large, irregular protrusions with distinct valleys and grooves, indicating a coarsened surface. For the combined treatment group (AG), a unique topography was observed, characterized by a wave-like appearance with defined peaks and valleys in an overall erosive pattern. The G Multiprimer group displayed a uniformly coarse appearance with faint grooves and blotchy surface coatings. In contrast, the control group (without surface treatment) exhibited a homogeneous, relatively flat surface with multiple grooves typical of a machined finish.

### 3.3. Surface Elemental Analysis Using EDAX (Figure 2)

Coupling SEM with EDAX allowed for elemental characterization of the specimens. All samples primarily consisted of zirconia, oxygen, and aluminum. Notably, the AA group and the combined AA with G-multiprimer group exhibited higher aluminum content, accompanied by correspondingly lower zirconia levels, than the non-AA groups. This is likely attributable to the aggressive roughening during AA, which causes material loss and leaves behind remnants of aluminum oxide particles.

Comparison of aluminum using one-way ANOVA test shows that the mean value of AG (2.463333) is highest, followed by AA (2.15), G-multiprimer (0.886667) least in control (0.653333). This difference is statistically significant with a test value of 34.923 and *p*-value of <0.001.

### 3.4. Failure Analysis (Table 2 and Figure 3)

A compound zoom microscope (40× magnification) was used to classify the failure modes as adhesive (A), cohesive (C), or mixed (M). The failure pattern in the C–NS and G-multiprimer (G–NS groups, regardless of saliva contamination, along with the saliva-contaminated AG–S group, predominantly exhibited adhesive failure. In contrast, 90% of the AA–S group demonstrated cohesive failure, while mixed failures were predominantly observed in the AA-NS and G–NS groups. Chi-square analysis confirmed that there was a significant difference in the failure mode distribution among the eight groups.

### 3.5. AFM and Surface Roughness

Representative 2D and 3D images obtained through AFM analysis of specimens subjected to different surface conditioning methods are depicted in Figure 4.

The AFM analysis showed that the roughness value of AA was highest, followed by G-multiprimer, AG, and least in control. This can be correlated to the SEM anning electron microscopy images. The control group showed a relatively smooth surface with a small quantity of irregular granular protrusions and pits scattered on the surface of zirconia ceramic. Due to the impact and abrasion of the alumina particles, larger irregular protrusions and pits were visible in the AA group. Multiprimer group presented more uniform pits and needle-like protrusion. Comparison of Ra, Rq, and Rmax values (using one-way ANOVA test) shows that the mean value of AA is highest, followed by G-multiprimer, AG is least in control. This difference is statistically significant with *p*-value of <0.001.

## 4. Discussion

This study examined the influence of three distinct surface treatment strategies, along with saliva contamination, on both the microstructure and bond strength between zirconia dental ceramics and SAC. The bond strength was quantified using a µSBS test, while the impact of the surface treatments on zirconia's microstructure was evaluated using SEM supplemented with EDAX and AFM. The failure modes were subsequently characterized using a compound microscope.

Since the different surface treatment strategies and salivary contamination had an impact on the µSBS of zirconia to resin cement, the null hypothesis was rejected. In the saliva-contaminated group, the µSBS was highest in the G–S, followed by AG–S, AA–S, and C–S, respectively. The results of this study are in agreement with previous studies done by Manso et al. [29], Murillo-Gomez et al. [30], and Ugur et al. [38]. The greater SBS with G-multiprimer, which contains both 10-MDP and γ-MPTS, may be due to the stronger chemical interactions of the resin and zirconia in addition to their mechanical interlocking.

The acidic environment created by MDP and SAC increases its effectiveness by activating γ-MPTS, prevents its hydrolysis and provides a stronger bond [38].

MDP bonding to zirconia has been shown to occur due to the dual function of the MDP molecule, including a hydroxyl group at one end of the phosphate group, which forms a bond with zirconia, and a saturated carbon at the other end, causing additional polymerization with unsaturated carbon in the matrix. It may be inferred that the incorporation of primer would benefit the bonding of zirconia with resin cement without the need for AA, even in difficult clinical scenarios where salivary contamination is difficult to control. This could also potentially reduce microcracks by AA. In addition, roughness produced by AA could increase adsorption of saliva, thereby affecting its bond strength. There have been conflicting reports in literature about the effect of AA on bond strength [31]. Increase in surface roughness on account of airabrasion does not necessarily influence the bond strength in linear proportion, as the adhesive and resin cement may not be able to flow entirely in the deep crevices created by roughness, further leading to a weaker bond of the zirconia with the resin cement [40].

In the NS contaminated groups, the µSBS was highest in specimens AG–NS, G–NS, C–NS, and AA–NS group, respectively. This suggests that, without contaminant interference, the primer can infiltrate effectively into the grooves created by AA, thereby enhancing both mechanical interlocking and chemical bonding [32, 33, 36, 41].

Comparison between saliva and NS-contaminated groups with different surface treatments revealed better results without any contamination. These findings are in agreement with previous studies [32, 33, 36, 41].

Saliva contamination has the potential to leave an organic adhesive coating on the bonding surface of restorative materials, which proves challenging to remove even with water and isopropanol cleaning methods [27]. There was a statistically significant difference between the control groups, as well as a combination of AA and the G-multiprimer group. These results provide insights that working in a moisture-free environment would yield the best results in terms of long-term bonding.

Nonuniform stresses arise at the bonding interface, extending into both the substrate and the resin cement, thereby resulting in cohesive or mixed fractures. The groups that showed adhesive-type failure were the control and G-multiprimer groups, irrespective of saliva contamination, and AA–S and G–S groups. This type of bonding failure can be explained by the absence of surface treatment on zirconia and the presence of saliva contamination. This may be attributed to the lack of chemical bonding to the ceramic surface, caused perhaps by the presence of an insulating contamination layer that was not removed by the alcohol cleaning procedure in the contaminated groups [33].

In addition, in vitro testing conditions may have contributed to the larger number of adhesive failures in the Multiprimer group despite having adequate SBS. The resin cement in contact with the zirconia disc specimen covered less than a mm in dimension while the blade used to shear the samples had a greater dimension. Therefore, unlike clinical scenarios, adhesive failure may be seen predominantly even when bond strength is greater in in vitro experiments, such as ours.

A cohesive type of failure was seen in 90% of AA– saliva group. This indicates that AA as a surface treatment could provide a good adhesive bond between zirconia and resin cement, irrespective of saliva contamination. Mixed type of failures was seen predominantly in the NS–AA and NS–G group, although bond strength in G -multiprimer was higher. There was a significant difference in the pattern of mode of failure in the eight groups. Representative SEM images of the groups supported these results. According to the EDAX findings, all the samples were composed of zirconia, oxygen, and aluminum. It was noted that the AA group and combination of AA and G-multiprimer group have more quantity of aluminum than other groups. This can be due to remnants of the aluminum oxide sand particles. The presence of aluminum element as confirmed by EDAX, is consistent with similar findings by Zhang et al. [42] and Paes et al. [43]. Furthermore, the zirconia content of these groups is less than the non-AA groups. It can be due to aggressive roughening of the surface during AA, leading to material loss and damage to the surface of zirconia. AA could also lead to the transformation of the crystal structure from the tetragonal to monoclinic phase [44].

AFM provided a quantitative, three-dimensional assessment of surface topography. The roughness parameters (Ra, Rq, and Rmax) measured by AFM confirmed that AA produces the deepest surface irregularities, which enhance primer penetration and mechanical interlocking. These quantitative measurements, along with the SEM findings, highlight the crucial role of surface morphology in optimizing resin cement adhesion. In the past few years, the pretreatment of zirconia surfaces was researched in depth to improve bonding with cement and make it more effective as well as long-lasting. It has been seen that airborne particle abrasion led to the creation of microfractures and changes on the restoration surface, leading to a reduction in zirconia's mechanical properties and their bonding durability. On the contrary, previous studies have shown that resin cements containing 10MDP, when used in conjunction with airborne particle abrasion, yield significantly enhanced bond stability and retention [45]. Magne et al. [45] studied the use of Z-PrimePlus on zirconia ceramics and concluded that there was an increase in the bond strength between them and various resin cements. A similar conclusion was drawn from a study conducted by Zandparsa et al. [46] and Go et al. [47], who concluded that combining the use of Z-Prime Plus and air-borne particle abrasion led to the improvement of bond strength between zirconia and resin cement.

The use of the G-multiprimer for enhancing the bond of resin cement and zirconia and its comparison with other surface treatments are scarce and are studied in this research. Additionally, the effect of saliva contamination on the surface-treated zirconia on its bonding with resin cement has been assessed. According to the study conducted, it can be concluded that surface treating zirconia with a combination of AA and G-multiprimer improves the bonding of resin cement on zirconia.

Our findings extend current knowledge of zirconia surface treatment by demonstrating that even in the presence of saliva contamination, the combination of AA and G-multiprimer application can enhance resin cement bonding. This nuanced insight may help refine clinical protocols for these materials.

The present study evaluated the µSBS between the zirconia and SAC non-MDP containing resin cement. Microshear testing, when compared to macro tests, requires only a small bonding area, leading to a uniform distribution of stresses and consequently yielding more dependable results. Many studies have utilized MDP containing self-adhesive cement in addition to surface treatment of zirconia with MDP-containing primers. This may have a larger impact on the bond strength between surface treated zirconia and cement than otherwise. Another notable limitation of this study is that only a single primer formulation (G-multiprimer) was evaluated. Future studies should explore additional primer types and formulations to determine whether similar or enhanced bonding improvements can be achieved.

Furthermore, in shear tests, it's crucial to recognize that the results primarily serve the purpose of comparing and ranking different materials and techniques. Further investigation into various types of zirconia and cements is warranted, given that variations in the chemical and physical properties of SACs could impact the bonding mechanism and durability when bonded to zirconia ceramic [48, 49].

Clinical Relevance: The combined treatment of AA and G-multiprimer application significantly increases resin cement bond strength to zirconia. This protocol is especially beneficial in clinical situations where moisture control is challenging, potentially leading to improved restoration durability and better long-term outcomes.

## 5. Conclusion

Within the limitations of this study, the combination of AA and G-multiprimer application yielded improved bonding of zirconia to SAC, suggesting a promising protocol for durable adhesion under controlled, moisture-free conditions.

## Figures and Tables

**Table 1:** Comparison of microshear bond strength (µSBS) of saliva and nonsaliva groups in each of the techniques using independent *t*-test.

Groups	Saliva	*N*	Mean	Std. deviation	*t*	df	*p*-Value
Control	Saliva	10	0.860	0.715	−2.536	10.219	**0.029**
	Nonsaliva	10	3.130	2.739			

Air abrasion	Saliva	10	3.146	2.615	0.201	18	0.843
	Nonsaliva	10	2.961	1.267			

G-multiprimer	Saliva	10	5.929	2.274	−0.01	18	0.992
	Nonsaliva	10	5.940	2.299			

Air abrasion + G-multiprimer	Saliva	10	4.973	0.996	−5.447	18	**<0.001**
	Nonsaliva	10	8.063	1.492			

*Note*: The *p* value is statistically significant when in bold.

**Table 2:** Mode of failure crosstabulation with chi square test summary.

Group	Adhesive (%)	Cohesive (%)	Mixed (%)
CS	100	0	0
CNS	90	0	10
AS	0	90	10
ANS	30	10	60
GS	90	0	10
GNS	80	0	20
AGS	90	0	10
AGNS	0	0	100
Total	60	12.5	27.5

**Table 3:** Roughness values in each test group (in nm).

Group	Ra (µm)	Rq (µm)	Rmax (µm)
C	0.156	0.204	2.215
AA	0.402	0.510	4.208
GP	0.280	0.362	2.881
AAGP	0.272	0.343	2.502

## Data Availability

The data that support the findings of this study are available upon request from the corresponding author. The data are not publicly available due to privacy or ethical restrictions.
